# Spatial tumor immune microenvironment phenotypes in ovarian cancer

**DOI:** 10.1038/s41698-024-00640-8

**Published:** 2024-07-18

**Authors:** Claudia Mateiou, Lavanya Lokhande, Lan Hoa Diep, Mattis Knulst, Elias Carlsson, Sara Ek, Karin Sundfeldt, Anna Gerdtsson

**Affiliations:** 1 Department of Pathology and Cytology, Institute of Biomedicine, Sahlgrenska Academy at University of Gothenburg, Gothenburg, Sweden; 2 Department of Immunotechnology, Lund University, Lund, Sweden; 3 Department of Obstetrics and Gynecology, Sahlgrenska Academy at University of Gothenburg, Gothenburg, Sweden

**Keywords:** Cancer microenvironment, Ovarian cancer, Ovarian cancer

## Abstract

Immunotherapy has largely failed in ovarian carcinoma (OC), likely due to that the vast tumor heterogeneity and variation in immune response have hampered clinical trial outcomes. Tumor-immune microenvironment (TIME) profiling may aid in stratification of OC tumors for guiding treatment selection. Here, we used Digital Spatial Profiling combined with image analysis to characterize regions of spatially distinct TIME phenotypes in OC to assess whether immune infiltration pattern can predict presence of immuno-oncology targets. Tumors with diffuse immune infiltration and increased tumor-immune spatial interactions had higher presence of IDO1, PD-L1, PD-1 and Tim-3, while focal immune niches had more CD163 macrophages and a preliminary worse outcome. Immune exclusion was associated with presence of Tregs and Fibronectin. High-grade serous OC showed an overall stronger immune response and presence of multiple targetable checkpoints. Low-grade serous OC was associated with diffuse infiltration and a high expression of STING, while endometrioid OC had higher presence of CTLA-4. Mucinous and clear cell OC were dominated by focal immune clusters and immune-excluded regions, with mucinous tumors displaying T-cell rich immune niches.

## Introduction

Ovarian carcinoma (OC) is the most lethal gynaecological cancer, with a 5-year survival rate of less than 50%^1^. Standard treatment includes a combination of surgery and systemic therapy^2^. Despite advances with aggressive cytoreductive surgery, approximately 70% of patients recur within three years^3^, with chemoresistant cancer that is typically incurable. The 5-year relative survival rate for stage III-IV invasive OC, which includes the majority of patients, is currently less than 30%. PARP inhibitors are approved for treatment of platinum-sensitive, recurrent OC, and newly diagnosed advanced OC with BRCA1/2-mutations and homologous recombination deficiency (HRD)^4^. Still, PARP inhibitors have limited long-term efficacy in the eligible group because of diverse resistance mechanisms.

Existing treatments could potentially be complemented with immunotherapeutic approaches for improved response rates. Immune checkpoint blockade has however shown modest effect in OC, and no FDA approved immunotherapeutic intervention is yet available, despite increasing evidence of immune infiltration playing a pivotal role for both initiation, progression, and chemotherapy resistance in OC^5^. While the association of immune infiltration and prolonged survival of OC has been recognized since long^6^, the heterogeneous immune response in OC may explain the failure of immunotherapy trials in unselected populations, and highlights the need for new strategies to improve the stratification of OC patients^7^. Immune infiltration has been correlated to survival in different studies^5^, demonstrating the vast potential for patient tailored strategies based on high-resolution immune profiling in OC.

OC can be grouped into five main histotypes, of which high-grade serous OC (HGSC) is the most prevalent (80%) and most studied in terms of immune infiltration. The less common subtypes include low-grade serous OC (LGSC), endometrioid, mucinous and clear cell OC. A unifying classification of Type 1 and Type 2 OC is sometimes used, where Type 2 are more aggressive, TP53-mutated tumors of chromosomal instability, which are near-ubiquitously HGSC^8^, and Type 1 are primarily LGSC, endometrioid, mucinous, and clear cell OC, commonly characterized as low-grade, indolent tumors with frequent alterations in cell signalling pathways^8^. While the characteristics of the different histotypes have been recently defined at genomic and transcriptomic levels^9–11^, variation in tumor immune microenvironment (TIME) and presence of immuno-oncology targets have not been extensively reported to date, particularly for the Type 1 histotypes. More defined immune profiles associated with the OC subtypes could aid patient stratification for immunotherapy eligibility.

Inflamed ovarian tumors with intra-epithelial compartment T-cells have been shown to have a favourable outcome, and it was recently shown that for BRCA1/2 mutated HGSCs, increased proximity of CD8+ and CD4 + T-cells to Ki67+ tumor cells was associated to improved prognosis^12^. These studies have demonstrated that prognostic information can be harnessed from the spatial distribution of immune cells. Studies of immune microenvironments in solid tumors are however still frequently based on subjective classification to stratify tumors as e.g., inflamed, excluded or immune-cold based on H&E or CD3 stains^13^. Approaches for reproducible and objective scoring of spatial immune infiltration patterns are needed^14^. With recent advances in image analysis and cell segmentation approaches, spatial statistics which better reflect heterogeneous tumor-immune topologies compared to e.g., average distance between tumor and immune cells^15^, can be collected and integrated in models for patient stratification. In addition, multiple immune phenotypes and molecular interactions have been shown to affect the outcome of OC, and the need for spatially resolved analysis of both tumor and stroma/immune compartments, with simultaneous measurements of multiple TIME markers, has been indicated in recent studies^16–19^. Thanks to the current emerge of novel digital pathology tools for multiplex, in-situ tissue analysis^20–22^, such analysis can now be achieved.

Here, we have used GeoMx Digital Spatial Profiling (DSP) of distinct spatial TIME niches, complemented with image-based analysis for spatial statistics that could aid in more objective and reproducible scoring of immune infiltration. We identified immuno-oncology targets associated with spatial tumor-immune topologies and assessed variation in immune infiltration patterns and TIME signatures across OC subtypes.

## Results

### Study workflow

**Fig. 1:**
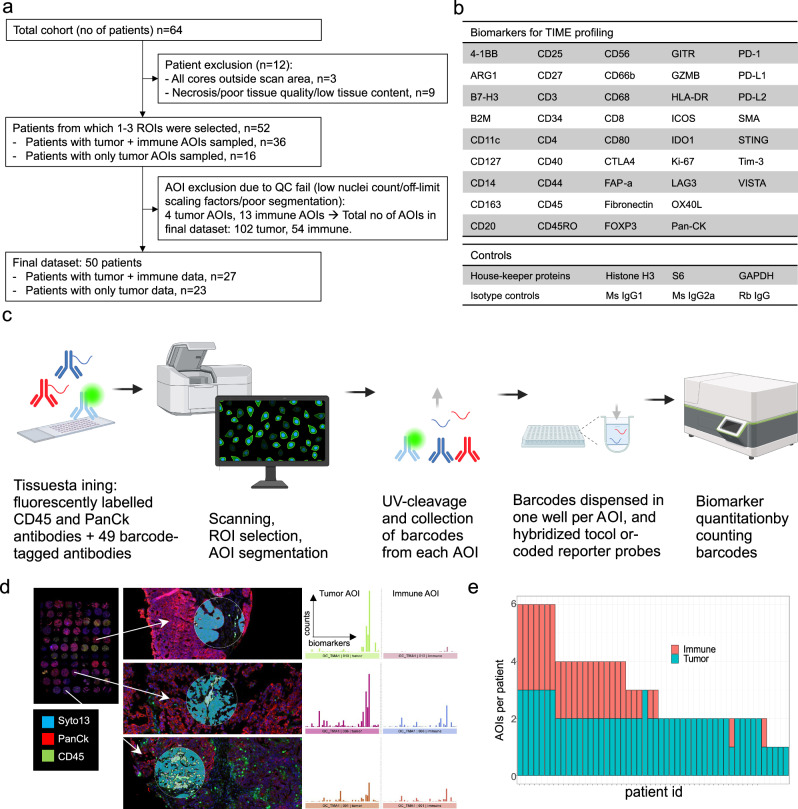
Experimental overview. **a** Sample inclusion. From a total of 64 patients, 12 were excluded due technical or quality reasons. Of 52 tumors deemed evaluable, 36 were estimated to have sufficient immune infiltration (> 20 CD45+ cells per ROI (Region of Interest)) for ROI segmentation into tumor (PanCk+ Syto13 + CD45-) and immune (CD45+ Syto13+ PanCk-) AOIs (Areas of Illumination). Following DSP (Digital Spatial Profiling) quantification of antibody-bound probes, 17 AOIs failed QC (quality control) process based low nuclei count, control normalization factor > 3 (indicating low probe binding), or poor segmentation. The final dataset consisted of 156 AOIs from 50 patients, with matched tumor and immune AOIs from 27 patients, and tumor only AOIs from 23 patients. **b** Specificities of antibodies used for tumor immune microenvironment (TIME) profiling. **c** Experimental workflow. The DSP technology includes staining FFPE (formalin-fixed paraffin-embedded) samples with panels of fluorescently labelled and barcoded antibodies. Immunofluorescence visualization through scanning is used to guide selection of ROIs. Upon exposure of UV light, barcodes are cleaved off, aspirated and dispensed in a microwell plate. Collected barcodes are hybridized to color-coded probes which are quantified in the nCounter instrument. **d** ROIs representing insignificant, focal and diffuse immune infiltration were selected from two TMA slides with 3x1mm cores per tumor (red=PanCk, green=CD45, blue=Syto13). Each ROI was segmented into AOIs of tumor (PanCk + , CD45-, Syto13 + , teal masks) and, when possible (> 20 immune cells per ROI), immune (CD45 + , PanCk-, Syto13 + , lime masks), for separate quantitation of biomarkers. Graphs show raw counts of biomarkers for tumor and immune AOIs, for the respective ROIs. **e** Data was collected from 50 patients, of which 27 had sufficient immune content per ROI for sampling of both immune and tumor AOIs. The number of AOIs collected per patient varied from 1-6.

From two TMA slides with a total of 64 OC tumors (Table 1), TIME phenotypes of 50 tumors (Fig. 1a) could be analyzed using DSP (Fig. 1c). IF staining of PanCk (epithelial cells), CD45 (immune cells) and Syto13 (nuclei) was guiding selection of 1-3 ROIs per patient (Fig. 1e), selected as representative areas of the TIME in each tumor (Fig. 1d). ROIs were segmented into tumor (PanCk+ Syto13 + CD45-) and, when possible, immune (CD45+ Syto13+ PanCk-) AOIs for separate quantification of 49 biomarkers (Fig. 1b). ROIs with < 20 immune cells were only segmented for tumor content. Following quality control filtering, the final data consisted of 156 AOIs from 50 patients (Fig. 1a). Of these, 27 tumors had sufficient immune infiltration for reliable quantification, enabling parallel profiling of tumor and immune AOIs (Fig. 1e). For the remaining 23 patients, only tumor AOIs were profiled.

**Table 1:** Patient cohort

	Total cohort *n* = 64	Tumor data *n* = 50	Tumor+Immune data *n* = 27
Age at diagnosis (years)			
Median	59	57	54
Min	21	25	28
Max	87	87	87
Malignant/Borderline/Benign			
Malignant	53	43	26
Borderline	7	6	0
Benign	4	1	1
Stage			
I	32	24	14
II	5	4	2
III	20	19	8
IV	3	2	2
NA (benign)	4	1	1
Histology			
HGSC	25	23	14
LGSC	5	5	3
Endometrioid	12	6	4
Mucinous	6	5	2
Clear cell	5	4	3
Benign/borderline	11	7	1
Type			
Type 1	28	20	12
Type 2	25	23	14
NA (benign/borderline)	11	7	1
Progression-free survival (yrs)			
Average	8.4	8.0	7.6
Min	0.5	0.5	0.6
Max	15.8	15.8	15.7
Response to therapy			
Complete response	42	34	19
Partial response	4	4	2
Stable disease	1	1	1
Progressive disease	1	1	1
Not reported	16	10	4
Progression during follow-up			
Yes	24	22	14
No	31	23	10
Not reported	9	5	3
Cause of death			
Ovarian cancer	16	15	9
Ovarian cancer treatment	1	1	0
Other	2	1	1
NA (alive at end of follow-up)	27	20	8
Not reported	18	13	9

The majority of biomarkers were normally distributed (Supplementary Fig. 1A). No significant difference was observed between the two TMA slides, which had been stained in the same batch, but scanned, collected and hybridized on two different occasions (Supplementary Fig. 1B). Immune AOIs had generally lower biomarker counts, owing to lower cell numbers and smaller areas of immune segments compared to tumor segments (Supplementary Fig. 1C). To avoid excessive data transformation, data was divided into immune and tumor, for separate processing and analysis. Normalization was performed by linear scaling to the geometric mean of GAPDH and S6, which showed the highest correlation among the housekeeper proteins (Supplementary Fig. 1D). Strong correlation between background (isotype control) level and nuclei count was neutralized by the normalization (Supplementary Fig. 1E).

### Classification of immune infiltration and tumor-immune spatial distribution

**Fig. 2:**
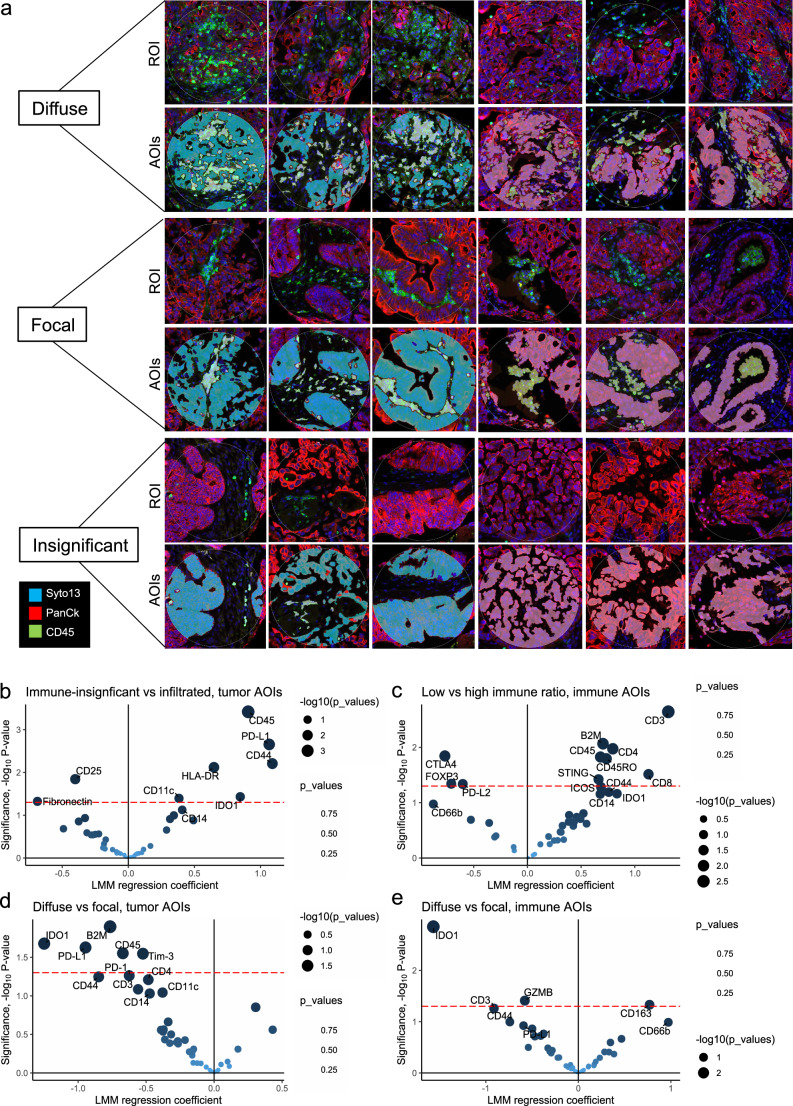
Spatial patterns of immune infiltration. **a** Six representative ROIs are shown for each of the spatial phenotypes; diffuse, focal and insignificant immune infiltration, three from each of the two TMA slides. Boundary of circular 300 µm diameter ROIs are shown on top, with segmented tumor/immune or tumor only AOIs below (red=PanCK, green = CD45, blue = Syto13). The segmentation masks are colored in teal (tumor) and lime (immune) for TMA1, and in pink (tumor) and lime (immune) for TMA2. **b** Tumor and immune protein signatures in spatial phenotypes of immune-insignificant and infiltrated tumors. Tumor segments in regions of insignificant immune infiltration had higher levels of CD25 and Fibronectin, while infiltrated tumor regions had higher CD45, PD-L1, HLA-DR, CD44, IDO1 and CD11c. **c** Immune segments from regions with low immune cell ratio were higher in CTLA-4, FOXP3 and PD-L2, while regions with high immune cell ratio were higher in CD3, beta-2-microglobulin (B2M), CD4, CD45, CD45RO, CD8, CD44, and STING. **d** Regions with diffuse immune infiltration had tumor segments that were higher in IDO1, PD-L1, B2M, CD45, and Tim-3, compared to regions with focal immune infiltration. **e** Immune segments of diffuse infiltration patterns were higher in IDO1, granzyme B (GZMB) and CD3, while focal immune segments were higher in CD163. Differential expression was assessed through linear mixed models (LMM) with Patient ID as random effect, including only malignant tumors. Significance (-log10 *p*-value) was plotted against LMM regression coefficient. Red dotted line marks *p* = 0.05.

The presence of immune cells was highly variable across the samples, with a mean percentage of CD45+ cells ranging from 0.3% to 69% in the sampled tumor regions (Supplementary Fig. 2A). Overall immune presence showed no significant association to overall survival (OS, Pearson R = −0.17, *p* = 0.26) or progression-free survival (PFS, Pearson R = −0,18, *p* = 0.25). ROIs were annotated by visual review as having significant (“infiltrated”, *n* = 65) or insignificant (“insignificant”, *n* = 37) immune presence, based on CD45 staining. The majority of ROIs from infiltrated tumors could be segmented based on PanCk, CD45 and Syto13 into tumor and immune AOIs, while most tumors with insignificant immune infiltration were segmented into a tumor AOI only due to scarcity of immune cells. Expression profile of lineage markers varied largely across patients (Supplementary Fig. 2B). Immune infiltrated ROIs were further annotated by the spatial immune infiltration patterns as “diffuse” (*n* = 35) for immune cells dispersed among tumor cells, or “focal” (*n* = 30) for immune infiltration largely present as clusters in the tumor, by visually assessing whether immune cells were more closely interacting with other immune cells (=focal clusters) or with tumor cells (=diffuse infiltration) (Fig. 2a).

To complement visual scoring, we used image analysis to generate objective and reproducible metrics of spatial relationship between tumor and immune cells from ROI images. To this end, we developed a workflow adapted to the 20X ROI immunofluorescence, multilayer OME-TIFF files which can be exported from the GeoMx software. Following image processing, cell segmentation and classification based on PanCk and CD45 were performed. Based on our ROI annotations, immune infiltrated tumors could be discriminated from immune insignificant tumors using ratio of immune cells over total cell count (*p* = 1.7e-11, Supplementary Fig. 3). Samples with under or over-estimated immune ratios compared to the visual scoring were found to have highly dense tumor areas and weak CD45 staining, or high CD45 staining background, respectively (Supplementary Fig. 3), which highlights the need for quality tissue and staining for semi-automated scoring by image analysis.

Graph network analysis was applied to assess tissue topology and used to calculate spatial statistics features related to cell-cell clustering and tissue architecture (listed in methods) based on a set distance threshold. Although ratio of immune cells was the strongest discriminant of infiltrated vs ignored tumors, it did not differ between regions annotated as having focal and diffuse immune infiltration (*p* = 0.21) (Supplementary Fig. 4A). Instead, group degree centrality (gdc) and tumor cell cluster co-occurrence ratio (ccr) were shown to better capture differences in immune infiltration patterns reflecting diffuse versus focal classification. Gdc here represents the fraction of tumor cells that are connected to at least one immune cell within a set distance, and tumor ccr is the frequency of connections between tumor cells compared to randomized data with equal number of tumor/immune nodes. Gdc and ccr were significantly different between regions annotated as having focal and diffuse infiltration for both distances (30 and 50 pixels) used as threshold, however stronger discrimination was observed at 30 pixels (=12 µm), (*p* = 0.007/*p* = 0.047 and *p* = 0.002/*p* = 0.017), for gdc and ccr at 30 px/50 px, respectively, Supplementary Fig. 4A).

### Tregs and fibronectin are associated with immune exclusion

Spatial phenotypes were compared using linear mixed models (LMM) with patient as random effect, to account for patient dependency and multiple ROIs being sampled per patient. The tumor protein profiles were skewed towards diffuse/infiltrated samples, due to that the majority of markers were immune related and thus not expected to be present in tumor segments devoid of immune cells. Still, tumor segments annotated as having insignificant immune infiltration were shown to display higher relative levels of CD25 and Fibronectin (Fig. 2b). LMM were also generated to predict features derived from the image analysis, which showed that areas with low immune cell ratio (< 0.2) had immune segments that were higher in CTLA4, FOXP3 and PD-L2 (Fig. 2c). Taken together, these analyses suggest that the presence of Tregs, as indicated by CD25, FOXP3 and possibly CTLA4, as well as Fibronectin is associated with immune exclusion. In comparison, infiltrated regions showed higher levels of activated lymphocytes, markers associated with antigen-presentation, and checkpoints PD-L1 and IDO1 (Fig. 2b).

### IDO1, PD-L1 and Tim3 are present in areas of diffuse immune infiltration, while focal immune niches are high in CD163

Areas annotated as having diffuse immune infiltration showed higher levels of MHCI (beta-2-microglobulin, B2M) and checkpoints IDO1, PD-L1, PD-1 and Tim-3 in tumor AOIs (Fig. 2d), and higher levels of T-cells (CD3) and Granzyme B in immune AOIs (Fig. 2e). Similarly, the graph network analysis showed that areas with high gdc, i.e., a high proportion of closely interacting immune and tumor cells, were high in T-cells, lymphocyte activation markers (CD44, CD40, CD80), dendritic cells (CD11c), but also immune suppressive markers PD-L1, Tim-3, IDO1, and VISTA (Supplementary Fig. 4B, C). In contrast, focal immune niches had higher levels of CD163, and tendency (*p* = 0.10) towards higher level of CD66b (Fig. 2e), suggesting a higher proportion of immune suppressive myeloid cells (M2 macrophages and possibly neutrophils) located in tumor-adjacent focal clusters. Significantly higher immune segment levels of CD163 were also associated with low gdc (Supplementary Fig. 4C).

**Fig. 3:**
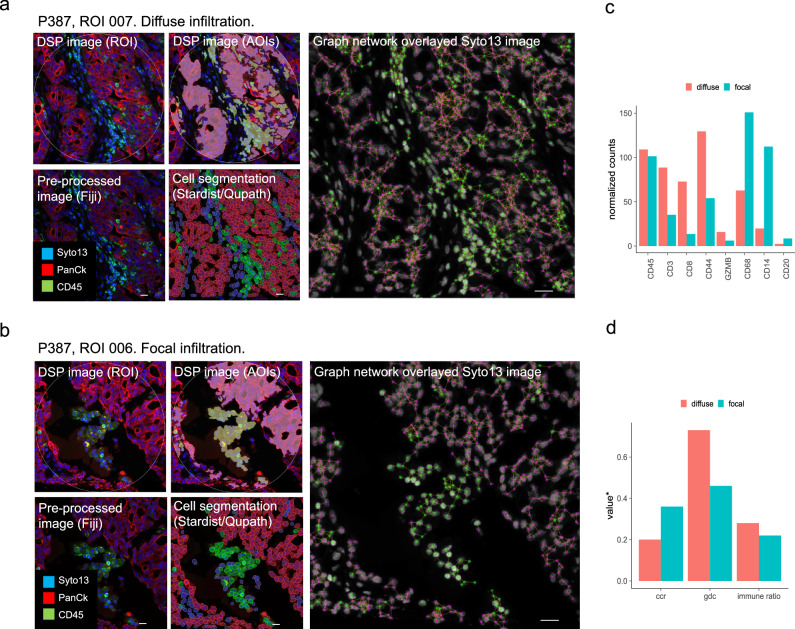
Combined image analysis and digital biomarker profiling exemplified for diffuse and focal immune infiltration regions in one HGSC patient. Immune infiltration of (**a**) diffuse and (**b**) focal patterns was identified in different cores of the same tumor (patient P387). Upper panels: Boundary of ROI (left) and segmentation into immune and tumor AOIs (right) in the DSP analysist (red=PanCk, green=CD45, blue=Syto13). Bottom panels: corresponding FIJI pre-processed ROI image (left), and DL-based segmentation and cell classification in QuPath (right). Right panels (larger image): Graph networks overlayed grey scale Syto13 ROI image. Nodes are colored in pink for tumor and green for immune. Connections within a 30-pixel (=12 µm) distance from centre of each node are displayed and were used to calculate spatial statistics. Scale bars represent 20 µm. **c** Normalized counts of selected biomarkers quantified in immune AOIs of diffuse and focal immune infiltration regions in tumor of P387. CD45 was similar in both AOIs. Diffuse immune infiltration was higher in CD3, CD8, CD44, and GZMB, and focal immune infiltration was higher in CD68, CD14 and CD20. **d** Spatial statistics derived from image analysis of the P387 tumor. Immune cell ratio was similar in diffuse and focal immune infiltration. Gdc was higher and tumor ccr lower in diffuse compared to focal infiltration. *the same y-axis scale are used for different spatial parameters; ccr scores, gdc scores and immune/(tumor+immune) ratio, respectively. ccr values have been scaled with a factor of 0.1 to enable visualization in the same plot.

In one HGSC tumor, ROIs had been identified in areas of both diffuse (Fig. 3a) and focal (Fig. 3b) immune infiltration, from two different tumor cores. ROIs from this patient (P387) were used to demonstrate the image analysis workflow from DSP ROI selection and segmentation into AOIs to image processing, cell detection and classification, and graph network analysis (Fig. 3a, b). Immune segment CD45 level (Fig. 3c) and ROI immune cell ratio (Fig. 3d) were similar in the two, again showing that the overall level of leukocyte presence could not discriminate between immune infiltration phenotypes. As expected, image analysis showed that gdc was higher in the ROI of more diffuse infiltration, while tumor ccr was higher in the ROI of more focal infiltration (Fig. 3d). These structural phenotypes were associated with ROI-specific differences in immune profiles, with diffuse immune infiltration showing stronger expression of CD3, CD8, CD44 and GZMB; while focal immune infiltration was higher in CD68, CD14, and CD20. Thus, in this particular tumor, diffuse immune infiltration showed a higher presence of activated T-cells while focal immune infiltration was enriched for macrophages and B-cells.

### Diffuse, focal and insignificant immune infiltration is observed across OC histotypes

**Fig. 4:**
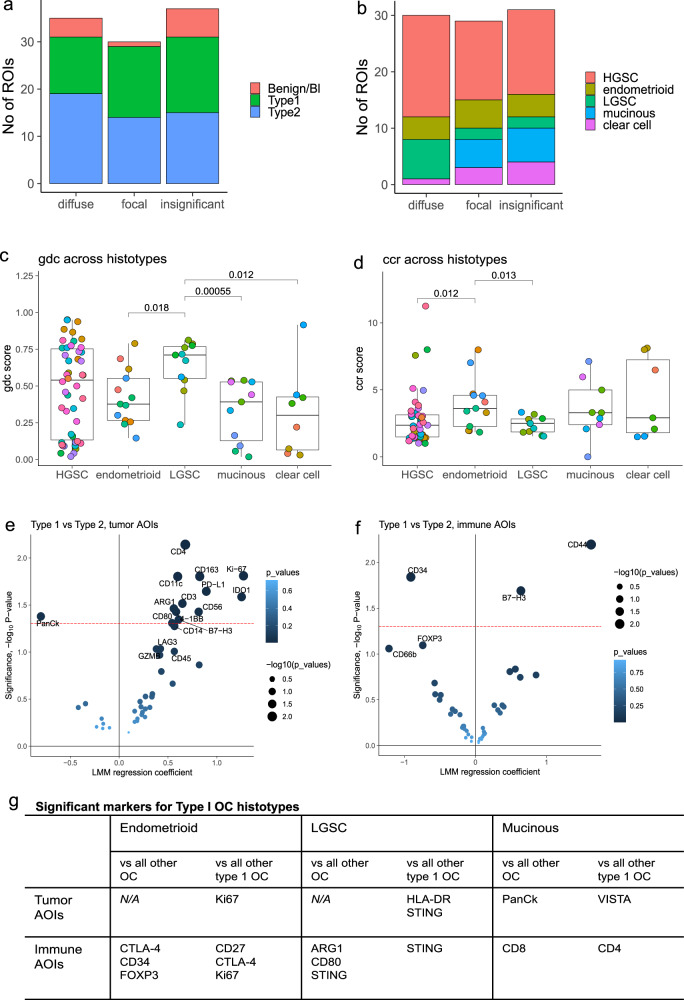
Tumor immune phenotypes in OC subtypes. **a** Diffuse, focal and insignificant immune infiltration were present in Type 1 and Type 2 malignant OC, as well as in benign and borderline samples. **b** Distribution of diffuse, focal and insignificant immune infiltration ROIs across histology subtypes of OC (malignant samples only). **c** gdc and (**d**) ccr across histotypes. ROIs are colored by patient IDs. Boxplots display median value (center line), first (lower hinge) and third (upper hinge) quartiles. Whiskers extend to the largest and smallest values, respectively. **e**, **f** Linear Mixed Models (LMM) with Patient ID as random effect and Type 1/2 as fixed effect to identify differences in immune infiltration between Type 1 and Type 2 OC in (**e**) tumor AOIs, and (**f**) immune AOIs. Only malignant tumors were included. Significance (-log10 p-value) were plotted against LMM regression coefficients. Red dotted line marks *p* = 0.05. **g** Biomarkers upregulated in low grade histotypes of OC as compared to all other samples and to other Type 1 histologies, respectively. Comparisons were made using LMM with Patient ID as random effect and histology as fixed effect. Biomarker significantly higher in each histotype are listed. Clear cell carcinoma is not included as no biomarkers were identified for that histotype.

Diffuse, focal and insignificant immune infiltration were represented in ROIs from both benign/borderline and malignant tumors. Among malignant tumors, all three spatial phenotypes were observed in both Type 1 (low-grade) and Type 2 (high-grade) tumors, although the frequency of diffuse immune infiltration was marginally higher in Type 2 OC (Fig. 4a). Although limited by small subgroups, malignant tumors were further stratified into respective histotype. Type 2 OC were exclusively high-grade serous carcinoma (HGSC), and Type 1 OC included low-grade serous carcinoma (LGSC), endometrioid, mucinous and clear cell carcinoma. Both immune-infiltrated and immune-insignificant regions were observed and sampled across all subtypes. For HGSC and endometrioid tumors, diverse infiltration patterns were seen, while LGSC mainly showed diffuse immune infiltration patterns. In contrast, focal or insignificant immune infiltration dominated mucinous and clear cell ROIs (Fig. 4b. In line with this, gdc was generally higher in the serous subtypes (Fig. 4c), indicating a higher frequency of tumor-immune interactions in particularly LGSC, while tumor ccr was higher in low-grade histotypes (Fig. 4d), significantly so for endometrioid OC.

### Type 2 OC shows stronger immune response and presence of multiple targetable checkpoints

To assess abundance of immune phenotypes and targetable markers in relation to OC histology and grade, the different subtypes of OC were compared using LMM with patient as random effect. Type 2 OC, which made up 46% of malignant samples in the final dataset, displayed higher level of Ki67, which was expected as HGSC in general has a higher proliferation rate. Higher PanCk was observed in tumor AOIs from Type 1 OC, which was also expected as low-grade tumors are more differentiated. In line with this, benign/borderline tumors had significantly higher level of PanCk compared to malignant tumors of all grades and stages (Supplementary Fig. 5). A number of phenotypic markers showed significantly higher expression in Type 2 tumor AOIs, including CD4, CD3, CD11c, CD163, CD80, CD56, and immuno-oncology targets PD-L1, 4-1BB, B7-H3, ARG1 and IDO1 (Fig. 4e). Immune AOIs had higher levels of CD34 in Type 1 OC, and close to significantly higher levels of FOXP3 and CD66b, while Type 2 immune AOIs were higher in CD44 and B7-H3 (Fig. 4f). Hence, Type 2 OC displayed an overall stronger immune response and presence of several immune suppressive targets. CD34, which was higher in Type 1 immune AOIs, is a marker of haematopoietic progenitor cells, but can also be expressed by subtypes of innate immune cells. Together with a higher expression of granulocyte marker CD66b and FOXP3, this indicates an, in general, more immature and regulated immune environment in Type 1 OC.

### Differential expression of key immune markers indicated in low-grade histotypes

Albeit limited by small groups, further subgrouping the malignant tumors into histotypes indicated key differences in immune phenotypes. Each histotype were compared to all other samples, as well as to other Type 1 samples only, and biomarkers significantly higher in each respective subgroup were listed (Fig. 4g). Endometrioid OC had immune segments that were significantly higher in CTLA-4 and FOXP3 (Fig. 4g, Supplementary Fig. 6A, Supplementary Table 1), which may indicate a generally stronger presence of Tregs compared to other histotypes and potential for CTLA-4 targeting. LGSC had significantly higher level of STING, suggesting a potential for agonistic immune stimulatory targeting. Mucinous tumors, which had immune infiltration frequently found in focal clusters, had significantly higher levels of CD8 and CD4 and low level of immune suppressive markers and checkpoints in the immune AOIs (Supplementary Fig. 6A), and tumor AOIs with higher expression of VISTA. The smallest group of clear cell OC (4 patients in final dataset) showed significantly lower levels of a vast number of biomarkers in both tumor and immune segments. The indication of an overall lower immune response in clear cell tumors however needs to be confirmed in larger cohorts. PD-L1 and IDO1 were more prevalent in HGSC compared to LGSC, mucinous and clear cell tumors, and on a similar level as endometrioid tumors (Supplementary Fig. 6B). In all, the differential expression of checkpoints between OC histotypes warrants validation in larger cohorts, to confirm whether abundance of immuno-oncology targets can be associated to specific subtypes.

### Spatial immune infiltration niches were not significantly associated with tumor stage and prognosis

The frequency of focal, diffuse or insignificant immune infiltration was similar across tumor stages (Supplementary Fig. 7A), and little variation in biomarker expression was observed as an effect of stage. LMM regression with stage as an ordinal scale fixed effect and patient as random effect identified immune AOI expression of LAG3 and 4-1BB as the only markers increasing with increased stage (Supplementary Fig. 7B). No biomarkers or spatial features were identified as having significant prognostic impact by Cox mixed effect (CoxME) models with patient ID as random effect and PFS as fixed effect. Among clinical features, only radical surgery (*p* = 0.007) and stage (*p* = 0.04) were significantly associated with PFS. Other known prognostic parameters, including Type 1/2 OC and patient age were non-significant, which highlights the stringency of the CoxME model. Type of immune infiltration was not identified as impacting survival based on CoxME. Kaplan-Meier (KM) analysis based on one value per patient, using consensus classification including all cores, confirmed that no difference in PFS was seen between tumors dominated by an immune-infiltrated versus immune-insignificant phenotype (*p* = 0.87, Supplementary Fig. 8A). Among infiltrated tumors, a trend (*p* = 0.086) of inferior PFS for tumors dominated by focal compared to diffuse immune infiltration was noted (Supplementary Fig. 8B). This was despite the (marginally) higher presence of focal infiltration in Type 1 OC, which have an overall better prognosis than Type 2, and is thus potentially an unfavourable effect of CD163+ macrophages, which were observed to a higher extent in the focal immune clusters.

## Discussion

The low success rate of checkpoint inhibitors for OC treatment is related to the notable variation in immune response in ovarian tumors, which highlights the need for TIME profiling to guide treatment options^23^. In addition, OC can be subdivided into several histotypes with different clinical presentations, and little is known about how the TIME and thus propensity for response to immunotherapy, varies across the subtypes. To this end, we assessed immune infiltration variation in relation to TIME topology and OC subtypes by measuring 43 immune related proteins in regions of interest representing tumor and immune areas with diffuse, focal, or insignificant immune infiltration patterns.

Immune infiltration was highly variable across samples, and the overall level of immune infiltration was non-prognostic. The spatial pattern of immune cells, i.e., distance and distribution in relation to tumor cells, had a higher impact on TIME molecular signatures than the overall level of immune infiltration. The proportion of CD45+ immune cells did not differ between areas of diffuse and focal immune infiltration, when generalizing over all sampled regions. However, areas of diffuse immune infiltration had more cytolytic activity as indicated by higher Granzyme B in immune segments, as well as higher tumor expression of targetable checkpoints PD-L1, Tim-3, and IDO1. Albeit non-significant (*p* = 0.09), patients with tumors dominated by focal immune clusters, had a tendency of inferior survival when compared to tumors dominated by diffuse immune infiltration. Focal immune clusters were more dominant in Type 1 OC, which in general are associated with improved survival. This may point to the highly unfavourable effect of CD163+ macrophages which were significantly higher in Type 2 vs Type 1 tumors, but also higher in focal immune clusters compared to diffuse immune segments. Tumor associated macrophages are in general the most abundant immune cell in the TIME^24^ and the ratio of M2-like over M1-like (CD163/CD68) macrophages have previously been associated to inferior survival in OC and other tumors^25,26^. There is still a lack of understanding to what extent the spatial distribution of CD163+ cells in the TIME effect outcome of different subtypes of OC. While our results indicate that checkpoint blockade targeting PD-L1 and/or Tim-3 and IDO1 should be more relevant for tumors dominated by diffuse immune infiltration, tumors with high presence of CD163+ focal immune clusters could potentially be subjected to TAM targeting therapies to increase susceptibility for checkpoint inhibitors or chemotherapy^27,28^.

Exclusion of cytotoxic immune cells from the TIME has been described as mediated by infiltration of tumor associated macrophages and/or Tregs^12^. By combining categorical and imaging-based stratification of tumors we here showed that immune-low/insignificant regions were higher in CD25, FoxP3, CTLA-4 and Fibronectin, suggesting that primarily Tregs (and potentially fibroblasts) are mediating immune exclusion. Finding new therapies for immune cold tumors remains a challenge and targeting of Tregs is associated with risks of severe side effects^29^. However, emerging evidence suggest that the effect of CTLA-4 antibody therapy may primarily be a result of depletion of Tregs rather than activation of effector T-cells^30^. Our comparison of subtypes of OC indicated a higher expression of Tregs in Type 1 tumors, and in this limited cohort, CTLA-4 was shown to be more prominent particularly in Endometrioid OC. Thus, while PD-L1 therapy may be more viable in HGSC with diffuse immune infiltration, CTLA-4 inhibition could potentially be an option for subtypes of Type 1 OC and immune cold tumors.

Classification of immune infiltration is commonly done by visual annotation, categorizing tumors as e.g., immune-hot, cold or excluded^13,14^. In an attempt to assess whether tumor-immune topology can be predictive of immune phenotypes and targets, we here selected and classified ROIs rather based on the tumor-immune topology, as either diffuse, focal or insignificant. Similar to previous studies, our classification was based on visual assessment and thus largely subjective. To complement the DSP analysis with improved segmentation algorithms and a more reproducible scoring of TIME toplogy, an image analysis workflow using OME-TIFFs exported from the GeoMx software, was developed. Deep learning-based single-cell segmentation and classification was performed in an external software, followed by graph network analysis. Spatial statistics including group degree centrality^31^ and cluster co-occurrence ratio^32^ are attractive features for semi-automated, objective scoring of TIME topology and cell-cell interactions, and correlated well with our annotations based on visual review. Our graph network analysis workflow could likely be improved by applying more advanced image deconvolution, including e.g. graph-based deep learning^33,34^. AI-based models have the potential for a more objective and automated stratification of tumors based on tissue tumor-immune topology and molecular information which potentially could be predicted even form low-plex immunofluorescence images^35,36^. These efforts hold promise for clinical implementation of spatio-molecular profiling to predict survival or response of therapy.

Importantly, this study is one of the first applications of spatial, molecular profiling of TIMEs across different histopathological subtypes of OC. Tumor segments in Type 2 OC (HGSC) were shown to have stronger infiltration of immune cells, including CD3, CD4, CD11c, CD163, CD56 and CD80, suggesting that both pro- and anti-inflammatory immune phenotypes are elevated inside the tumor nests compared to Type 1 OC. While CD4 was the most differentially higher expressed marker in Type 2, CD8 did not differ between Type 2 and Type 1 OC, which in this particular cohort likely was due to a prominent presence of CD8 also in the mucinous subtype. In addition, targetable and predominantly immune suppressive markers, PD-L1, IDO1, ARG1, 4-1BB, and B7-H3, were more abundant in Type 2 OC. HGSC has previously been shown to display a highly varied TIME^14,37^. Considering the molecular heterogeneity of HGSC, further stratification of this subtype based on e.g. BRCA1/2 mutations may be required to optimally predict propensity for different immunotherapy strategies. Indeed, previous studies have demonstrated that HRD status impacts the TIME of HGSC^17^, and that tumor-intrinsic mutational profiles are associated to specific immune evasion mechanisms^38^. Unfortunately, mutational profiling had not been performed on this retrospective cohort.

Grouping into Type 1 and Type 2 OC has been criticised for being overly simplistic, with Type 1 not capturing the heterogeneity of low-grade OC (here represented by low-grade serous, mucinous, clear cell, and endometrioid tumors). Recent studies have shown that non-serous subtypes not always cluster together based on molecular features^9^. In line with this, we could not identify a generic TIME profile for the low-grade tumors, with CD34 being the only significantly upregulated marker for the collected group of Type 1 tumors. Thus, profiling of the individual subtypes is likely more relevant for clinical implications. Although limited by low number of patients, our study indicated that there are key differences in TIMEs across the histotypes. For example, mucinous tumors displayed a more active immune response compared to the other low-grade carcinomas, with relatively higher levels of CD8 cytotoxic T cells and immune activation marker VISTA, while lower in immune suppressive markers (IDO1, FOXP3, CTLA4). In contrast, endometrioid tumors were indicated to have a stronger presence of Tregs (FOXP3 and CTLA-4), and tumor PD-L1 and IDO1 levels on par with HGSC. Interestingly, LGSC had high levels of STING, an immune stimulatory target which has been suggested an attractive therapeutic target in OC in general^39^, and which also previously was shown to be highly expressed in LGSC in particular^40^. The small set of four clear cell tumors showed an overall weak immune response relative the other histotypes. The low immune activation in endometrioid and clear cell OC compared to other histotypes is noteworthy considering that mismatch repair deficiency is frequent in these subsets^41,42^ and needs to be confirmed in larger cohorts.

We recognize that the limited tissue material provided on TMAs may be a confounding factor to capture TIME heterogeneity in a tumor^43,44^. The TMAs used in this study had been previously constructed from representative tumor areas, thus not with the purpose of profiling immune infiltration. We addressed potential bias by using TMAs with three cores per tumor and, whenever possible, sampling multiple ROIs per tumor. Importantly, this limited cohort was primarily used to assess whether immune biomarkers could be associated to patterns of immune infiltration in selected regions. On-going analysis of a larger cohort will serve to further establish spatio-molecular signatures associated to OC histotypes and prognosis.

The field of spatio-molecular profiling is only in its infancy but carries vast potential for guiding personalized treatments in the clinical setting. In this study, we showed that there are distinct differences in molecular phenotypes related to tumor-immune topology in ovarian carcinoma. Based on the protein signatures identified, patients with more diffuse tumor immune infiltration are more likely to benefit from targeting PD-L1/PD-1, IDO1 and/or Tim-3, while tumors with focal immune infiltration patterns showed increased presence of CD163+ macrophages and a preliminary worse outcome. These results demonstrate the potential value for stratification of tumors by spatio-molecular profiling for preselection for immunotherapy inclusion.

## Methods

### Clinical samples

A tissue microarray (TMA) of 64 surgically resected, mixed-histology OC tumors had been previously constructed from tissue collected between 2001 and 2010 at the Sahlgrenska University Hospital. The study complied with all relevant ethical regulations including the Declaration of Helsinki. Sample collection was approved by the Swedish Ethical Review Authority (reference 201–1545)^45^. The patients included received written and oral information in Swedish and signed the informed consent. Cohort characteristics are listed in Table 1. Whole biopsies were sectioned and stained with Hematoxylin (Histolab Products AB, Sweden). Three representative tumor areas were identified under the light microscope (Olympus BX45, Olympus Corporation, Tokyo, Japan), and three 1 mm cores were punched with a manual tissue microarrayer (Beecher MTA-1, Estigen,Tartu, Estonia) and re-embedded into a predefined position on a new, paraffin block. The TMA block was heated at 45 °C for 1 h, sectioned at 4 μm, mounted onto two slides, and stored at -80°C. Benign and borderline cases were excluded from analyses that compared spatial TIME niches, histologies and subtypes (Type 1 and Type 2 OC). Tumor regions could be sampled from 50 patients (77% of total cohort). Of these 50 tumors, 27 (54%) had sufficient immune infiltration for parallel tumor and immune niche sampling (Fig. 1a).

### Antibodies

The TMA was analyzed using GeoMx Digital Spatial Profiling (DSP, Nanostring, Seattle, WA, USA)^46^. 3-color immunofluorescence was used for visualization of cell nuclei (Syto13), tumor cells (PanCk) and immune cells (CD45) using the GeoMx Solid Tumor TME Morphology Kit v1.0 (Nanostring) and profiled using 49 antibodies (Fig. 1b) including the GeoMx panels for immune cell profiling (core panel, 18 targets and 6 controls), immuno-oncology drug targets (10 targets), immune activation status (8 targets), and immune cell typing (7 targets). The controls included negative isotype controls mouse IgG1, mouse IgG2a and rabbit IgG; and positive housekeeper controls Histone H3, S6, and GAPDH.

### Sample preparation

The GeoMx Protein Slide Preparation protocol was applied, using the DSP Protein Slide Prep Kit for FFPE. Briefly, TMA slides were baked at 60 °C followed by deparaffinization and rehydration (3 x 5 min CitriSolv, 2 x 5 min 100% EtOH, 2 x 5 min 95% EtOH, 2 x 5 min ddH_2_O); antigen retrieval for 15 min in 1xCitrate buffer, pH 6.0 at high pressure, high temperature; and washing for 5 min in 1xTBS-T. A hydrophobic pen was used to create a closed barrier on the slides. All incubations were performed in a black humidity chamber. Slides were blocked (Buffer W, 1 h, RT); incubated with antibody cocktail diluted in Buffer W (overnight, 4 °C); washed in 1xTBS-T (3 x 10 min); fixed with 4% PFA (30 min, RT); washed in 1xTBS-T (2x5 min). Finally, slides were stained with Syto13 (500 nM in TBS, 15 min RT), and dip-washed twice in 1xTBS-T.

### Sample processing

The two TMA slides (TMA1 and TMA2) were scanned and processed in the GeoMx instrument on two separate days. Immediately after scanning, 1-3 Regions of Interest (ROIs) were selected per tumor. Typically, one ROI was sampled per tumor core, and no tumor had more than two ROIs placed in the same core. All ROIs were circular with 300 µm diameter. ROIs were segmented into tumor (PanCk+ Syto13 + CD45-) and, when possible, immune (CD45+ Syto13+ PanCk-) Areas of Illumination (AOIs). Thresholds for segmentation were adjusted for each ROI. Guided by the segmentation masks, each AOI was sequentially exposed to UV-light to cleave off oligos coupled to antibodies that had bound in that specific segment and aspirate the oligos to separate wells in GeoMx collection 96-well plates (Fig. 1c). Aspirates were dried at 65 °C in a thermal cycler, rehydrated in 7 µL DEPC water and spun down. Probes were hybridized with GeoMx hyb codes according to the GeoMx DSP Protein nCounter Readout scheme, incubated overnight at 67 °C and stored at 4 °C. Hybridized products were pooled by columns into strip tubes, in volumes related to the total segment area collected according to the GeoMx protocol. Quantification was performed using the nCounter (Nanostring) system, followed by transfer of readout data back to the GeoMx instrument (Fig. 1d).

### DSP data processing and analysis

The GeoMx analysis suite was used for data quality control (QC), using default QC settings. This step also normalized the data to the positive hybridization controls, to adjust for inherent variability across the lanes of the nCounter cartridge used for probe quantification. Data was filtered (Fig. 1a) by assessing AOIs that were flagged in the QC process as having either low nuclei count (<20 cells) or high positive control normalization factor (>3), by visual inspection of scan images and by principal component analysis to check sample biomarker data in relation to distribution of dataset. Following QC and filtering, immune and tumor data were separated into two datasets in order to avoid scaling bias during biomarker signal normalization due to inherent differences in AOI size (immune segments were generally smaller than tumor segments) (Supplementary Fig. 1). Both immune and tumor data were (separately) normalized by scaling each AOI with the geometric mean of housekeeper proteins GAPDH and S6 for that specific AOI. Following QC, data was exported from the GeoMx analysis suite and imported to RStudio (2022.02.03) for downstream processing and analysis in R (R-4.1.2). Linear mixed models (LMM) with Patient ID as random effect were used to identify biomarkers differing between groups of samples, to adjust for patient dependency from sampling multiple ROIs per tumor, using the lmerTest package (3.1-3)^47^. The coxme (2.2-16) package was used for mixed effects models identifying biomarkers associated to survival. The survival (3.2-13) R package were used for Kaplan-Meier (KM) analysis. P < 0.05 were considered significant for all three types of tests (LMM, CoxME, KM).

### Image analysis

ROI tiff images were exported from the GeoMx analysis suite and preprocessed in ImageJ for splitting hyperstacks to individual images, performing background subtraction with rolling ball radius set to 100 pixels, and recombining to composite images. Processed images were imported to Qupath (v. 0.3.2). Cell segmentation was performed using a pretrained StarDist model (dsb2018_heavy_augment.pb) followed by training of two RTrees classification models, first for stroma and tumor (PanCk channel), and second for immune (CD45 channel). Models were combined into a composite classifier and applied on all ROI images. Cell class and coordinates were then exported from QuPath to python (3.10). The networkX (3.0) package was used for graph analysis, with weighted edges using Euclidean distance band using libpysal (4.6.2). Thresholds of 30 pixels (=;12 µm) and 50 pixels (= 20 µm) were applied. The original grey-scale tiff images were overlayed with the resulting network graph for visualization. Image derived spatial statistics were extracted from each ROI, including number of nodes per class, class ratio, degree centrality (fraction of tumor cells connected to immune cells within a given ratio)^31^, attribute assortativity coefficient (relative degree of connections to same class (e.g. immune) versus different class (e.g. tumor) cells)^48^, cluster co-occurrence ratio (ratio of connections between nodes compared to randomized data with equal number of nodes of each class) between cells within a class (tumor-tumor; immune-immune) and between classes (immune-tumor))^15^.

### Supplementary information

Supplementary Fig.s and tables

## Data Availability

The datasets used and/or analysed during the current study are available from the corresponding author on reasonable request.

## Code availability

The underlying code for this study is not publicly available but may be made available to qualified researchers on reasonable request from the corresponding author.
