# Supplementary material for: Spatial tumor immune microenvironment phenotypes in ovarian cancer
Source: NPJ Precis Oncol. 2024 Jul 18;8:148. doi: 10.1038/s41698-024-00640-8 (PMC11258306; doi:10.1038/s41698-024-00640-8)
Supplement: Supplementary file 1 — Supplementary Fig.s and tables [file 41698_2024_640_MOESM1_ESM.pdf]

Supplementary Figure 1

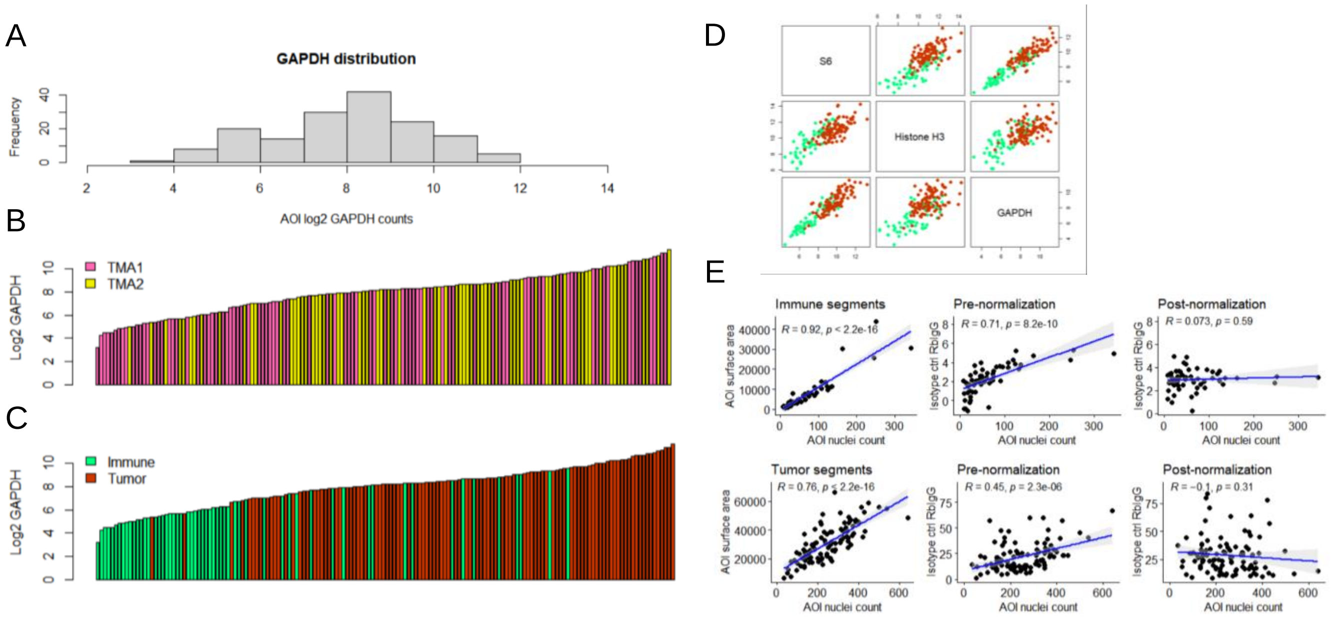

Supplementary Figure 1. Data preprocessing. A) Biomarkers generally demonstrated normal distribution, as exemplified by housekeeper protein GAPDH. B) No significant difference between TMA1 and TMA2 was observed, as demonstrated by GAPDH counts in a barplot sorted from lowest to highest AOI. C) Biomarker counts were systematically lower in immune segments compared to tumor segments due to general lower nuclei counts and AOI areas of immune segments compared to tumor segments, as demonstrated by GAPDH counts. D) Correlation between housekeeper proteins S6, GAPDH, and Histone H3. Immune AOIs in green and tumor AOIs in red. E) Top panels: immune AOI data, bottom panels: tumor AOI data. Left: Correlation between AOI surface area and nuclei count. Correlation between isotype control (Rabbit IgG) and AOI nuclei count for raw (quality control adjusted) data (middle panels) and post-normalization (right panels).

Supplementary Figure 2

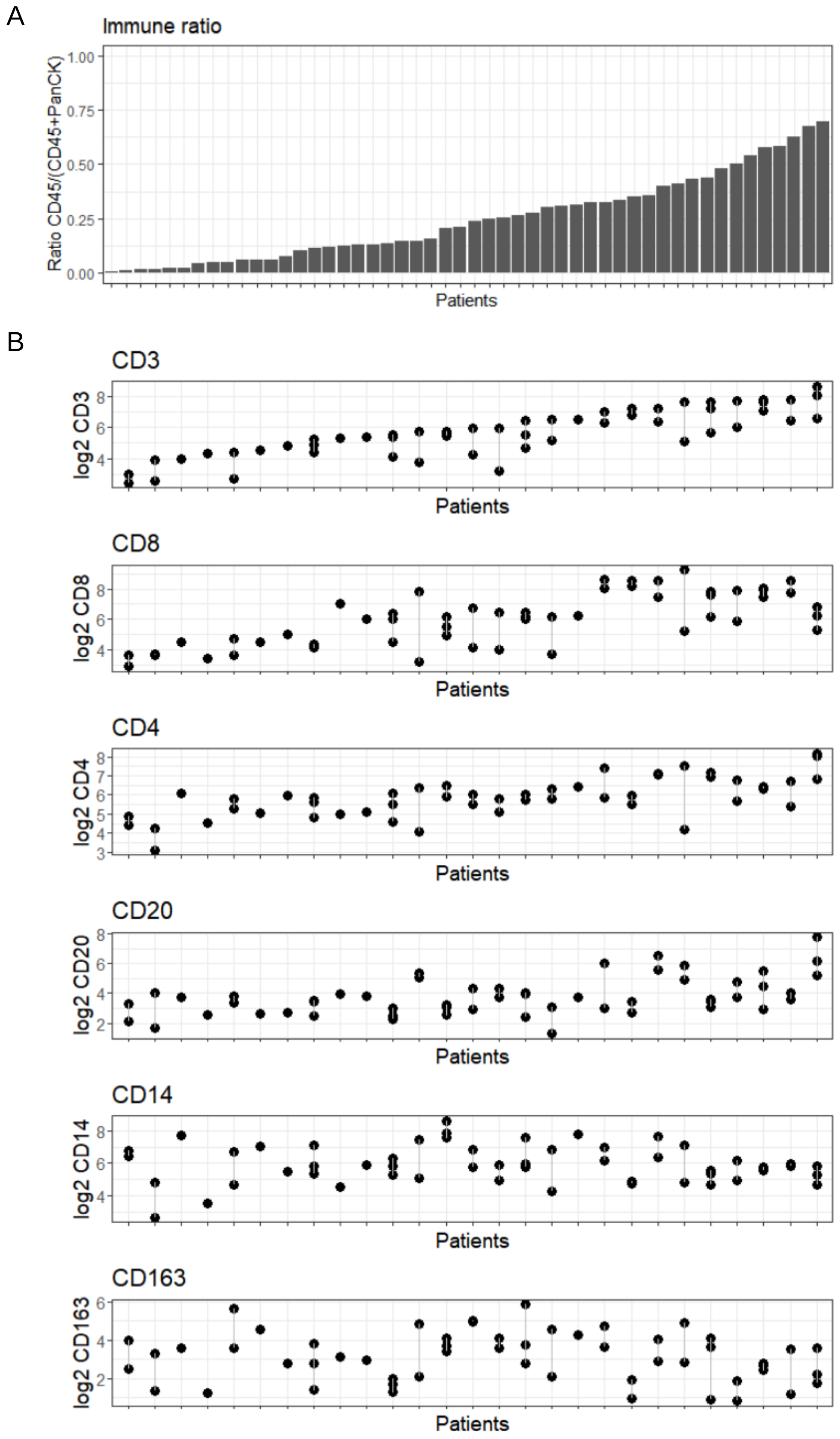

Supplementary Figure 2. Immune infiltration heterogeneity in OC. A) Proportion of immune cells per tumor as quantified by immunofluorescence image analysis of sampled areas. Average ratio of CD45+ cells over total count of CD45+ and PanCk+ cells were plotted per patient. B) Variation in DSP-quantified, normalized counts of selected lymphocyte and myeloid lineage markers in sampled CD45+ segments, all plotted in order of increasing CD3 value (highest AOI) for comparison across tumors.

Supplementary Figure 3

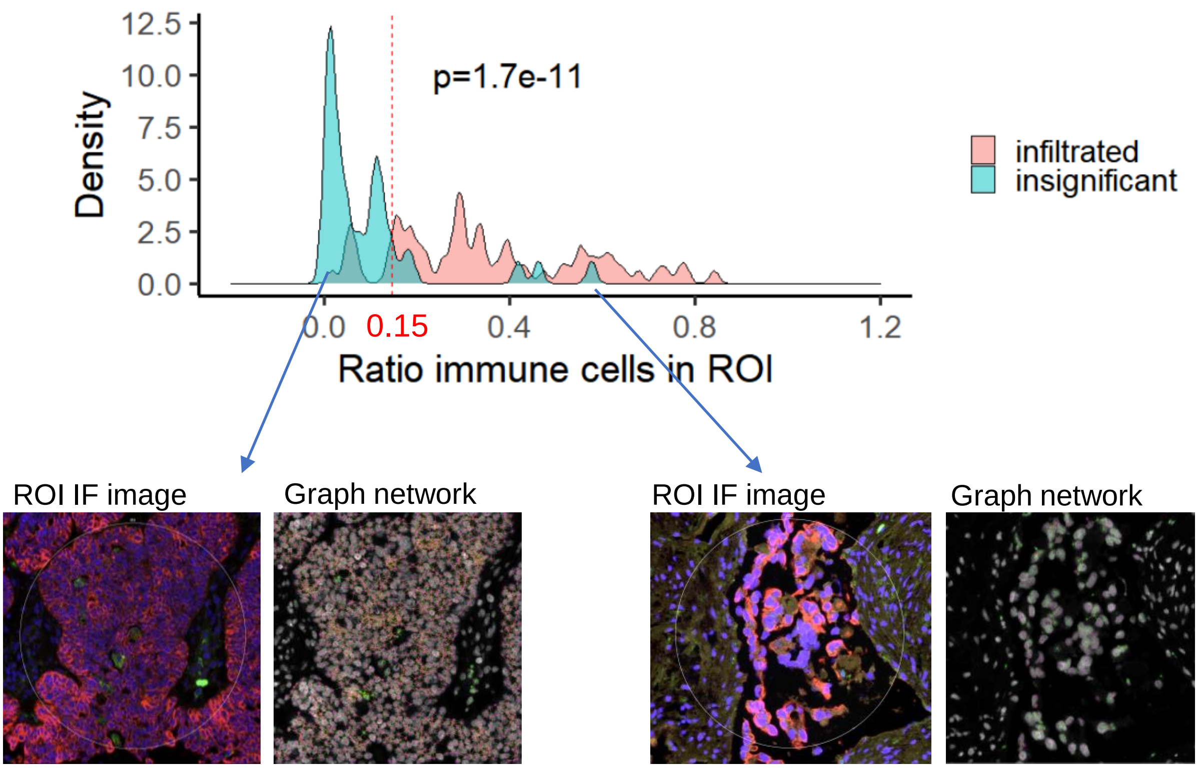

Supplementary Figure 3. Ratio immune cells over all cells as determined by image segmentation and classification of images exported from the GeoMx software. Each square image included the ROI in the center, and the area immediately surrounding the circular ROI. Thus, more cells were counted from the image analysis than were sampled from the corresponding ROI. An approximate immune cell ratio of 0.15 were found to best discriminate tumors classified as infiltrated vs insignificant, based on visual scoring. Outliers are exemplified: Left: ROI 012 on TMA1 classified as infiltrated despite a low immune cell ratio determined by image analysis, due to highly dense tumor area and weak CD45 staining. Right: ROI 022 on TMA2 classified as immune-insignificant. Here, the high CD45 staining background resulted in false positive scoring of immune cells in the image analysis.

Supplementary Figure 4

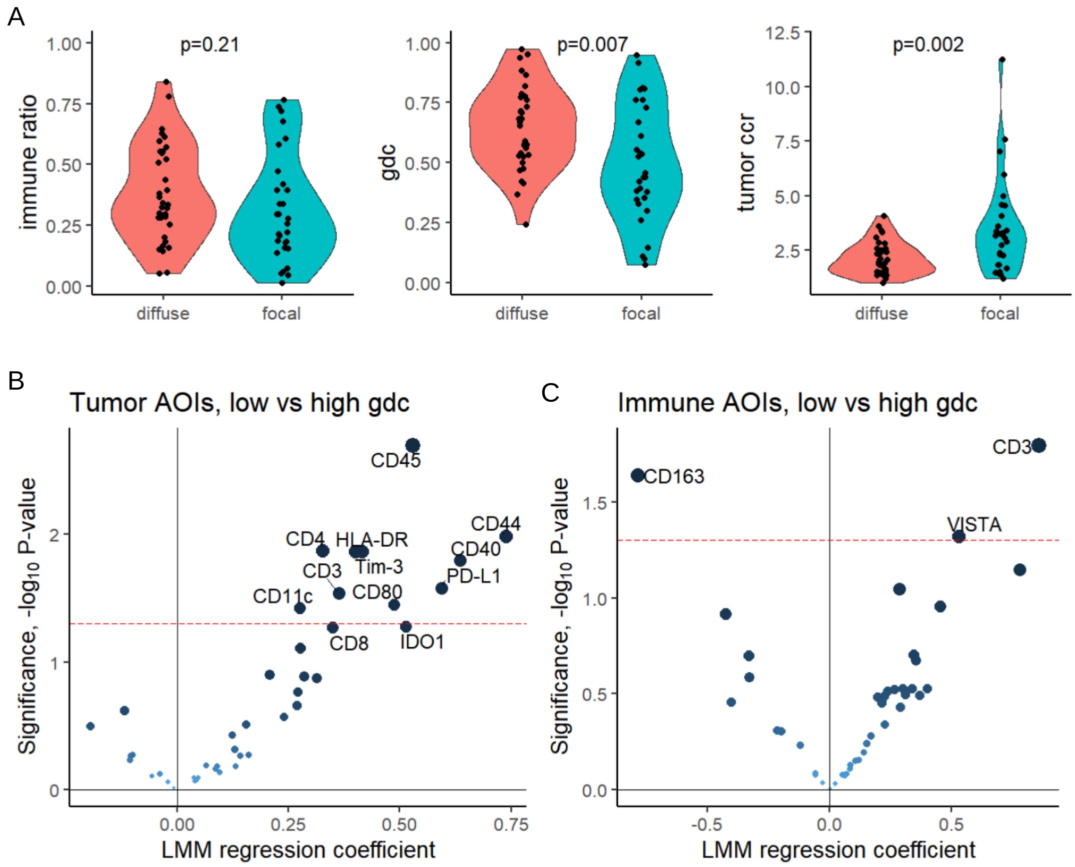

Supplementary Figure 4. Spatial features determined by graph network image analysis. A) Immune cell ratio (left), group degree centrality (gdc) (center) and tumor cluster co-occurrence ratio (ccr) grouped by ROI classifications as determined by visual evaluation of CD45 and PanCk staining patterns. B) Protein profiles of tumor segments and C) immune segments with low vs high immune cell ratio. D) Protein profiles of tumor segments and E) immune segments with low vs high group degree centrality (gdc). F) Protein profiles of tumor segments and G) immune segments with low vs high tumor-immune cluster co-occurrence ratio (ccr). Spatial features were determined for a 30-pixel (12 µm) distance from each cell. For gdc and ccr, sample were divided in high and low in equally sized groups. Differential expression was assessed through linear mixed models (LMM) with Patient ID as random effect. Significance (-log10 p-value) was plotted against LMM regression coefficient. Red dotted line marks p=0.05.

Supplementary Figure 5

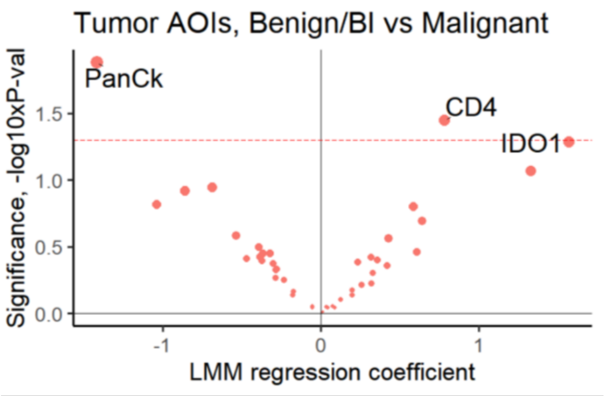

Supplementary Figure 5. Tumor-immune microenvironment phenotype in benign and borderline vs malignant tumors. LMM regression with patient as random effect, and benign/borderline/malignant as fixed effect. The number of patients and ROIs were for benign: 1 patient, 2 ROIs; borderline: 6 patients, 9 ROIs; malignant: 43 patients, 91 ROIs. Immune AOIs in malignant and benign/borderline could not be compared as only one immune AOI was collected from benign/borderline tumors.

Supplementary Figure 6

A

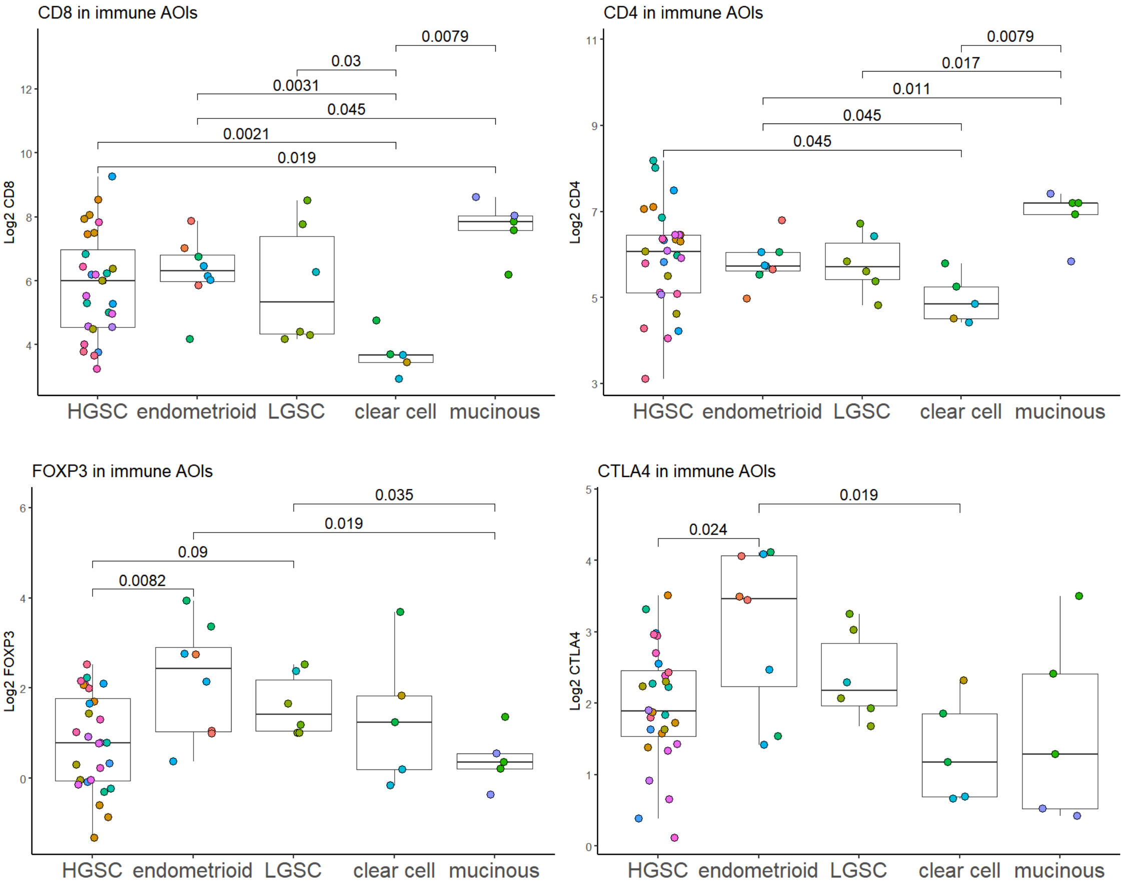

B

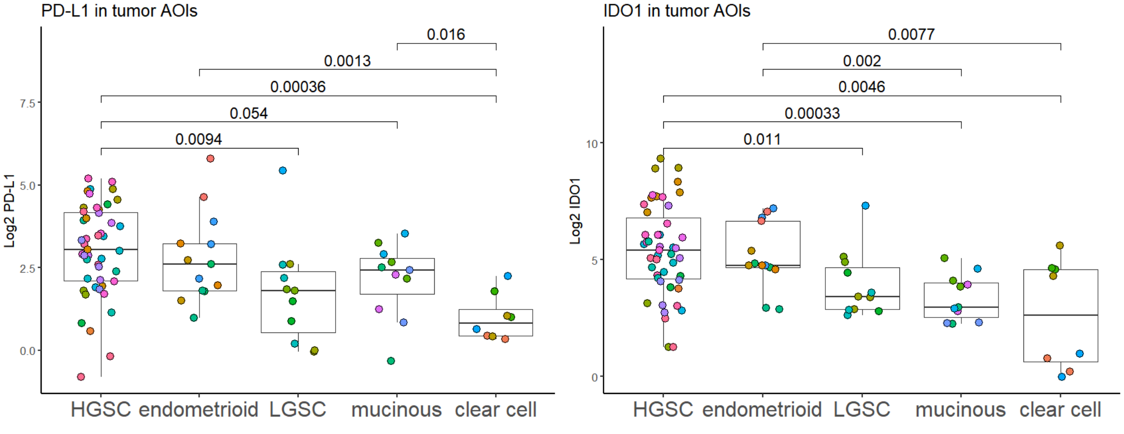

Supplementary Figure 6. Biomarker distribution across OC histotypes. A) immune AOIs: CD8, CD4, FOXP3 and CTLA4; and B) tumor AOIs: PD-L1 and IDO1. Wilcoxon p-values are shown for significant (p<0.05) different groups. Note that p-values were here calculated using all AOI data, including 1-3 AOIs per patient. Overlayed data points are colored by patient.

Supplementary Figure 7

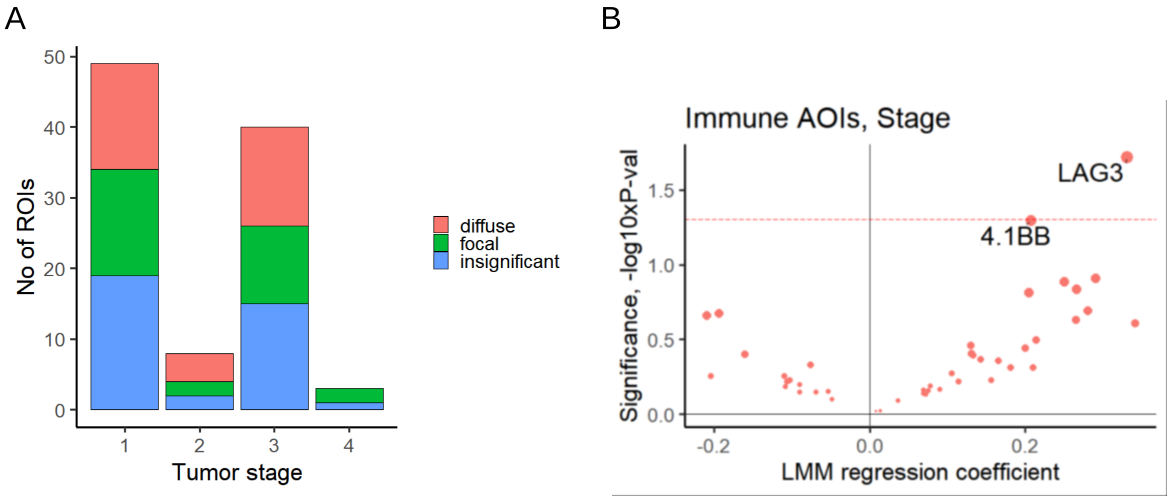

Supplementary Figure 7. Immune infiltration association to tumor stage. A) Number of AOIs per tumor stage. B) LMM regression with patient as random effect, and stage as fixed effect. Stage was annotated as stage 1, 2, 3, 4 (i.e. stage IA, IB, IC were all grouped into stage 1).

Supplementary Figure 8

A

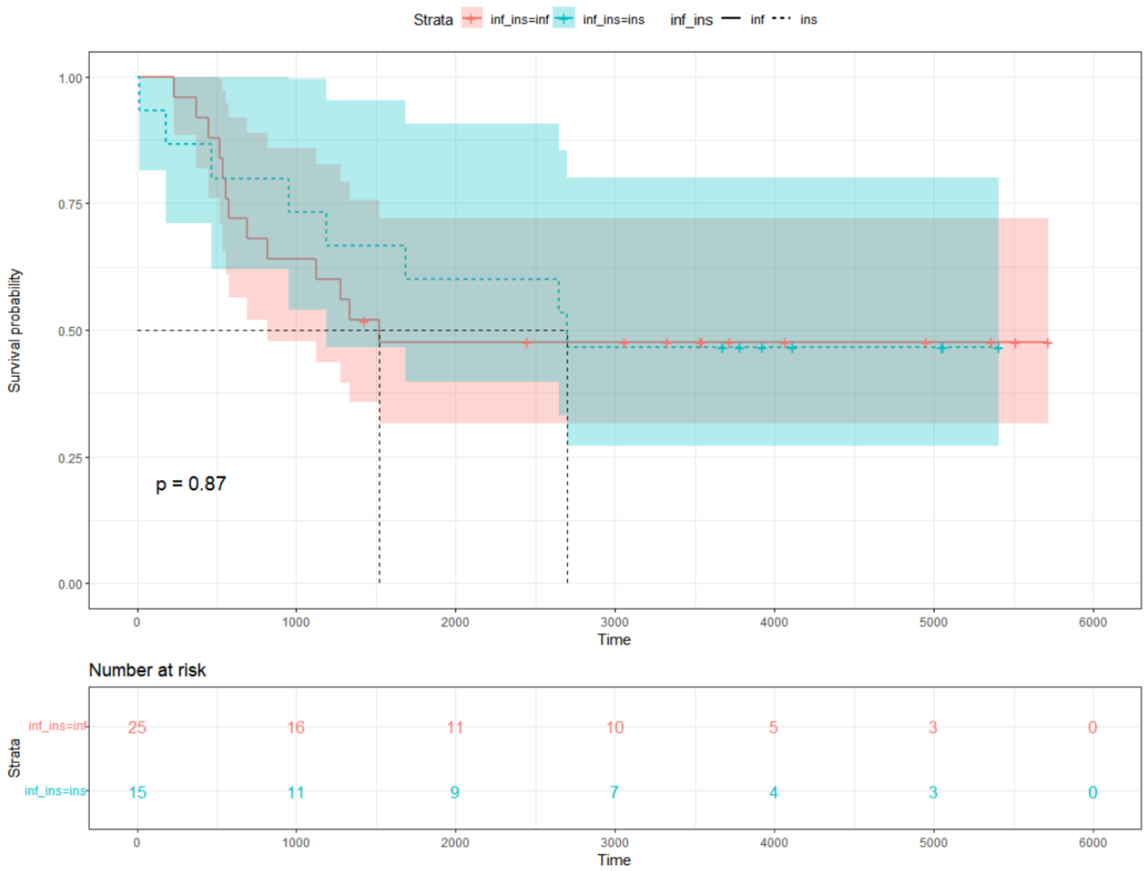

B

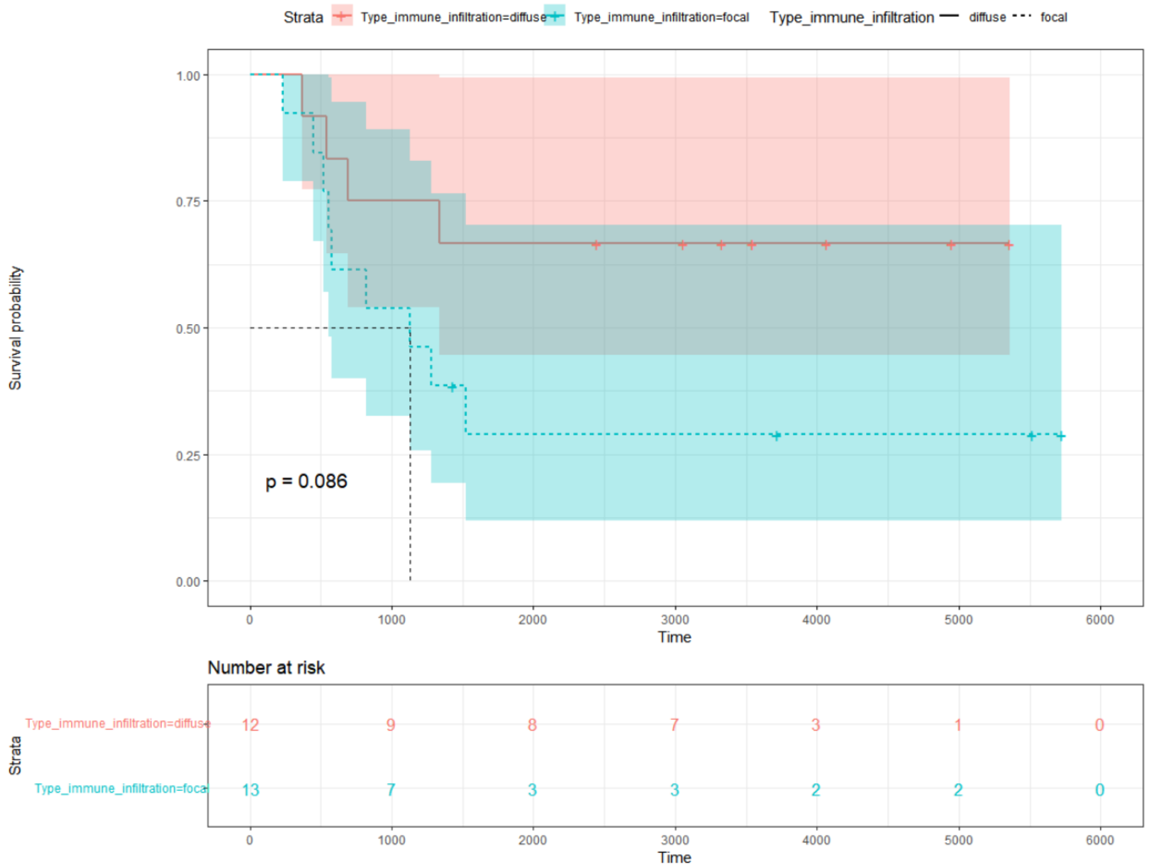

Supplementary Figure 8. Survival analysis by Kaplan-meier for patients annotated as having A) immune-infiltrated (inf, n=25) versus ignored (insignificant, ins, n=15) tumors; B) immune infiltrated tumors annotated as having either diffuse (n=12) or focal (n=13) immune infiltration.

Supplementary Table 1.

		Tumor AOIs				Immune AOIs			
		No of patients, ROIs*	Marker	P-value	Coeff	No of patients, ROIs*	Marker	P-value	Coeff
endometrioid	vs all other OC	6, 13	N/A			4, 8	CTLA-4<br>CD34<br>FOXP3	0.008<br>0.009<br>0.029	1.22<br>1.34<br>1.25
	vs all other type 1 OC		Ki67	0.012	1.98		CD27<br>CTLA-4<br>Ki67	0.004<br>0.023<br>0.036	1.26<br>1.30<br>1.15
mucinous	vs all other OC	5, 11	PanCk	0.009	1.56	2, 5	CD8	0.048	2.05
	vs all other type 1 OC		VISTA	0.023	0.87		CD4	0.017	1.32
LGSC	vs all other OC	5, 11	N/A			3,6	ARG1<br>CD80<br>STING	0.009<br>0.017<br>0.017	1.25<br>1.58<br>1.24
	vs all other type 1 OC		HLA-DR<br>STING	0.031<br>0.048	1.63<br>1.06		STING	0.006	1.25

*Supplementary Table 1. Comparison of OC histotypes using linear mixed models, inlucding p-values and LMM coefficients for all significantly (p<0.05) upregulated biomarkers in each of the low-grade histotypes, respectively. No biomarkers were found to be significantly upregulated in clear cell carcinoma. *No of patients and no of ROIs are listed for the specific histotype, i.e., the smalles group in the comparison.*
